# Multisensory integration and white matter pathology: Contributions to cognitive dysfunction

**DOI:** 10.3389/fneur.2022.1051538

**Published:** 2022-11-02

**Authors:** Jeffrey R. Hebert, Christopher M. Filley

**Affiliations:** ^1^Physical Performance Laboratory, Marcus Institute for Brain Health, University of Colorado School of Medicine, Aurora, CO, United States; ^2^Behavorial Neurology Section, Department of Neurology and Psychiatry, Marcus Institute for Brain Health, University of Colorado School of Medicine, Aurora, CO, United States

**Keywords:** sensory integration, sensory processing, white matter, cognition, multiple sclerosis, mild traumatic brain injury

## Abstract

The ability to simultaneously process and integrate multiple sensory stimuli is paramount to effective daily function and essential for normal cognition. Multisensory management depends critically on the interplay between bottom-up and top-down processing of sensory information, with white matter (WM) tracts acting as the conduit between cortical and subcortical gray matter (GM) regions. White matter tracts and GM structures operate in concert to manage both multisensory signals and cognition. Altered sensory processing leads to difficulties in reweighting and modulating multisensory input during various routine environmental challenges, and thus contributes to cognitive dysfunction. To examine the specific role of WM in altered sensory processing and cognitive dysfunction, this review focuses on two neurologic disorders with diffuse WM pathology, multiple sclerosis and mild traumatic brain injury, in which persistently altered sensory processing and cognitive impairment are common. In these disorders, cognitive dysfunction in association with altered sensory processing may develop initially from slowed signaling in WM tracts and, in some cases, GM pathology secondary to WM disruption, but also because of interference with cognitive function by the added burden of managing concurrent multimodal primary sensory signals. These insights promise to inform research in the neuroimaging, clinical assessment, and treatment of WM disorders, and the investigation of WM-behavior relationships.

## Introduction

In the investigation and conceptualization of human cognition, much attention has been focused on gray matter (GM) structures as the primary regions of interest. Key areas include the neocortices and the hippocampus, and subcortical GM structures including limbic nuclei, the thalamus, and the cerebellar GM (1–9). Gray matter structures important for memory, for example, include the hippocampus, thalamus, and prefrontal cortex [PFC; (10)], and the cerebellum is engaged in the mediation of attention, language, visuospatial processing, and executive function (8). These GM regions, however, do not act independently of each other, and instead, white matter (WM) tracts and GM structures operate in concert.

One of the critical functions of WM in the operations of cognition is the management of multisensory signaling (11). To effectively process sensory signals from the periphery and modulate cognitive behavior, structural interactions between GM regions are vital. White matter tracts comprise the anatomical basis for these interactions (10, 12), serving as a crucial foundation for bottom-up and top-down behavioral integration of multimodal sensory stimuli.

Humans constantly process sensory data from various independent sources, particularly from the visual, auditory, and vestibular end organs. Participation in daily life activities, which requires a continually adapting cognitive repertoire, typically occurs in ever-changing environments, where it is rare that a single sensory stimulus requires processing. Sensory input is thus typically multimodal and requires a complex network of cerebral regions that integrate sensory and cognitive systems. The thalamus, and the thalamo-cortical radiations by which it is connected to the sensory cortices, play key roles in both bottom-up processing that provides central integration of sensory signals from the periphery, and top-down processing that enables higher level behavioral regulation. However, other GM and WM structures are also involved in responses to multimodal sensory stimuli that require centralized weighting and re-weighting of sensory information. For example, when visual and vestibular stimuli are concurrently processed, the middle and inferior frontal gyri (13), the visual and vestibular cortices, the thalamus (14), and the posterior cerebellar vermis (15) are collectively activated and synchronized. The ability to simultaneously process and integrate multiple sensory stimuli is critical for normal cognition and effective daily function across the lifespan (16). The development and lifelong maintenance of cognition is dependent on unaltered multimodal sensory management and processing of convergent signals from several sensory systems, most notably vision, audition, and vestibular (17).

This review delves into the relationship between primary sensory processing and cognitive dysfunction in relation to WM and its pathology, in areas such as impaired attention, an integral function between bottom-up sensory-mediated selection and top-down control of sensory processing. Such relationships are an important focus in behavioral neurology, where much work has recently been done to interrelate attention, sensory integration, and white matter pathology (18, 19). The focus here will be on conceptualizing sensory integration-mediated cognition in terms of WM tract integrity, with an emphasis on three primary sensory modalities: visual, auditory, and vestibular. The contributions of WM pathology and related sensory processing deficits to cognitive impairment will be highlighted using a detailed consideration of these concepts in two common disorders with substantial WM pathology, multiple sclerosis (MS) and mild traumatic brain injury (mTBI). In these disorders, cognitive dysfunction in association with altered sensory processing may develop directly from slowed signaling in WM tracts and, in some cases, GM pathology secondary to WM disruption, or because of sensory overload from managing multisensory stimuli that results in added interference with normal cognitive processing.

## Sensory modality processing, networks, fiber tracts, and cognition

Supported by the underpinnings of postsynaptic potentials and synaptic integration models (20), conceptualizations of the interplay between sensory processing and cognitive function rooted in thalamo-cortical circuitry are evolving (11, 21–24). To begin, visual and auditory stimuli are processed within the thalamic lateral and medial geniculate bodies. These sensory nuclei are first-order structures that act as preliminary processers of sensory signals. This initial encoding serves as bottom-up conveyance of ascending sensory input to the primary sensory cortices for higher cortical processing and preliminary behavioral response (25). Concurrently, the thalamic reticular nucleus serves as the coupling mediator between the thalamus and primary sensory cortices through branched connections within bidirectional thalamo-cortical tracts. This arrangement serves to filter out redundant, superfluous stimuli, a process referred to as sensory gating, which leads to greater efficiency of brain response and behavioral modulation (11). Similarly, the pulvinar shares in this process of sensory gating by specifically modulating visual attention (26). Sensory data are further processed through transthalamic signaling streams that involve thalamo-cortical signal transference (27). These thalamo-cortical pathways serve to augment cortical function, forming the basis of top-down processing necessary for the development and modulation of cognitive domains such as attention and memory (22). This processing is necessary because visual and auditory stimuli are pragmatically conflicting, and unresolved competitive visual and auditory signaling leads to a greater load on higher-level top-down processing, which in turn increases demands on selective attention (28).

Multimodal sensory events result in the recruitment and activation of multiple brain regions beyond those required to process unimodal stimuli, leading to the added complexity of simultaneously processing and managing more than one mode of sensory stimulus. For example, when a person performs a visual function task, the addition of auditory signals during task performance requires the activation of thalamo-cortical and transthalamic pathways for completion of the visual task (29). Wolff and colleagues describe how temporal and spatial summation act to integrate separate unimodal inputs (e.g., visual, auditory) into a coalescent modulatory postsynaptic signal in the primary sensory cortices (11). This multimodal signal stacking enhances integration by collectively aggregating the different sensory modal signals to levels that reach the excitatory postsynaptic threshold necessary for cortical activity in structures such as the PFC, thus enhancing cognitive function (11). White matter tracts are the integral connections that tether GM structures together to form cognitive networks (30). Concurrent processing of auditory and visual signals has been linked to activity of the cingulo-opercular region [including the anterior insula, operculum, dorsal anterior cingulate, and thalamus; (31)], an important network for attention, processing speed, memory, sensory perception, speech, and language (32–36). Overlapping with the cingulo-opercular network, the cerebello-frontal network, consisting of other subnetworks including a cerebello-thalamic pathway, is related to cognitive processing speed (37). The cerebello-cortical network is also related to visual attention and working memory (38), and along with cerebello-thalamic and cerebello-pons subnetworks, is instrumental in auditory-mediated attention and memory (39). The fronto-parietal network is also important in that it helps mediate executive function, attention, memory, processing speed, and cognitive flexibility (34, 35, 40–43). In all of these networks, WM provides structural connectivity between GM regions.

In the central integration of multiple sensory stimuli, networks, and cognition, two prominent association tracts, the cingulum and the uncinate fasciculus, merit special attention. The cingulum lies on the medial aspect of the cerebral hemispheres and connects frontal, temporal, parietal, and occipital regions (44). Considered a component of the limbic system (45), the cingulum connects a variety of cortical and subcortical GM regions and forms the matrix of a major exchange route linking sensory processing and cognitive function. Cingulum projections, including thalamo-cingulate and thalamo-cortical fibers and the hippocampal cingulum, serve to augment bottom-up and top-down connections that process sensory stimuli and manage executive function, working memory, and attention (44, 46). Visuospatial function has also been mapped to the cingulum (47), and cingulum disintegrity is correlated with age-related decline in visuospatial performance (48). Abnormal fiber metrics in the cingulum have been found in persons with mild cognitive impairment who have memory, language, and visuospatial dysfunction (49).

The uncinate fasciculus, also a component of the limbic system (45), is a large association tract with a protracted maturation until 30 years of age or greater. The uncinate connects the PFC and the anterior temporal lobe, and thus plays a major role in emotional regulation (50, 51). This fasciculus enables cortico-limbic integration by way of PFC and amygdala connectivity (52), and, together with the cingulum and the thalamo-hippocampal-PFC circuit (53), is instrumental in linking cognition and sensory processing. A recent report from Shiotsu et al. showed that healthy young adults who are more sensitive to auditory stimuli have greater integrity of the uncinate fasciculus (54). This fasciculus is also associated with executive function and memory (55–60), creativity (61), and emotional intelligence (62). Age-related cognitive decline is related to reduced integrity of the uncinate fasciculus (63), and this phenomenon, coupled with altered sensory processing in aging (16), further supports the link between impaired multisensory processing, WM pathology, and cognition.

Although less thoroughly studied than the visual and auditory systems, the vestibular system is also critical for cognition. The physiology of the vestibular end organ is unique in that it delivers multiple distinct sensory inputs (e.g., angular acceleration, linear acceleration), rendering the central processing of vestibular signaling much less linear and more complex to process. Moreover, multiple classes of second-order vestibular neurons of the vestibular nuclei also receive input from visual and somatosensory systems and the cerebellum (64–68). There is no single vestibular-specific thalamic nucleus; instead, vestibular signals are processed by multiple thalamic nuclei, and vestibular-mediated potentials also project to other areas of the brainstem, and cerebellar and cortical regions (69, 70). Compared to visual and auditory modalities, relatively little is known about the central processing of vestibular signaling, including its WM connections and its impact on cognition (71). Nevertheless, with the diversified spread of vestibular signaling throughout the brain comes a variety of neuronal networking and processing demands, and behavioral responses that directly implicate cognitive function. It has been reported, for example, that vestibular signaling leads to responses in WM tracts relevant to cognition (e.g. cingulum) and GM [e.g., frontal cortex, hippocampus; (72)], and processing of vestibular signaling has been associated with multiple cognitive domains such as visuospatial function (e.g., spatial memory, navigation), attention, processing speed, memory, and executive function (73). Animal model and human research provide empirical results that link vestibulopathy and cognitive impairment, showing that advanced levels of vestibulopathy (e.g, bilateral involvement, severe hypofunction, extended duration) are associated with hippocampal atrophy and related cognitive impairment (74, 75). These findings have prompted the hypothesis that vestibulopathy may be a risk factor for the development of dementia including Alzheimer's Disease (75, 76). Moreover, age-related vestibulo-limbic-cortical pathway degeneration (75), may implicate WM pathology as a potential contributor to dementia pathogenesis.

## Altered sensory processing, white matter pathology, and cognitive dysfunction

### Multiple sclerosis

White matter tract demyelination is the signature neuropathology of MS and is thought to result from autoimmune-mediated inflammation that produces classic demyelinative plaques in the brain and spinal cord (77). When inflammation is more severe, axons are damaged in addition to myelin. Focal MS lesions can also occur in GM, although the primary pathology is demyelination and neuronal injury only develops later. This multifocal lesional pathology produces a wide range of clinical manifestations, including abnormal sensory processing in multiple modalities (78–80) and cognitive impairment (81).

Recent reports abundantly support deficient multisensory processing in MS. Giurgola et al. report that persons in the early stages of MS (e.g., relapsing-remitting disease) demonstrate abnormal multimodal processing involving concurrent visual and auditory signals, which may lead to cognitive impairment (82). Altered unimodal sensory processing and related cognitive impairment is also described in MS. For example, vestibular signal processing is impaired in patients with MS, and is associated with cognitive dysfunction, including slowed processing speed, impaired memory, and visuospatial dysfunction (83). Moreover, cognitive impairment related to modified visual signal processing is detectable early in the disease, and becomes more evident in later stages when impaired visual processing correlates with other deficits including slowed processing speed and poor memory (84).

In recent years, pathologic involvement of the thalamus has been linked with cognitive impairment in MS (85). Thalamic volume loss, for example, has been shown to predict cognitive impairment in the domains of attention, memory, and executive function (86). White matter connections of the thalamus are also damaged, and diffusion tensor imaging (DTI) has disclosed altered thalamic connectivity in association with cognitive loss; one study showed disruption of the thalamo-hippocampal-PFC network in early MS with cognitive impairment (86). Lower integrity of the cingulum and uncinate fasciculus has also been associated with cognitive impairment (87), and conversely, higher integrity of the uncinate has been shown to predict processing speed in MS (86). Recently, Fritz et al. found that lower diffusivity of the superior cerebellar peduncle and reduced volume of the superior and middle cerebellar peduncles related to slower processing speed in persons with MS (88).

The correlations between thalamic and cerebellar volume loss and cognitive impairment in MS raise the question of how a WM disease can so prominently affect GM. Many factors may be involved, but one answer may be that WM tract demyelination appears to precede GM atrophy. An important connection has recently been made between WM and GM pathology in MS by Lie et al., who found that GM degeneration occurs secondarily to WM damage (89). This phenomenon is most prominent in the early stages of the disease, whereas in later stages GM demyelination also contributes to atrophy (89). Lie et al. propose that both retrograde degeneration (backward from the damaged site toward the cell body) and anterograde or Wallerian degeneration (forward from the damaged site toward the axon terminal) contribute to GM atrophy (89). The thalamus appears to be susceptible to this phenomenon (89, 90), helping explain the link between thalamic damage and cognitive impairment. In the context of this review, observations of thalamic atrophy in MS (89) further elaborate the notion of altered sensory processing affecting cognition *via* WM pathology.

### Mild traumatic brain injury

Diffuse axonal injury (DAI), a result of shearing forces that damage multiple WM tracts, is the most important pathology of moderate-to-severe non-penetrating TBI (91), and is likely to be present in mTBI as well (92, 93). Mild TBI-related DAI often leads to widespread and disabling clinical manifestations, and whereas traditional thinking has maintained that post-mTBI symptoms typically resolve within 2–3 months, more recent reports suggest that symptoms can persist far longer in some individuals (94–96).

In particular, post-mTBI cognitive symptoms are more persistent than previously recognized (97). The reasons for this persistence are multifactorial, but a growing body of evidence is implicating DAI, and potentially chronic inflammation and later degenerative pathology, as important determinants of lasting symptomatology (98). Among many WM tracts vulnerable to mTBI, the cingulum and the uncinate fasciculus have both been shown to be damaged by DAI, and this form of injury predicts cognitive impairment (99, 100).

With respect to sensory function, DAI of prominent WM tracts is likely to further exacerbate post-traumatic cognitive sequelae by decoupling the interplay between sensory processing and cognitive function (101). Dysfunctional unimodal sensory processing of visual (102, 103), auditory (104, 105), and vestibular (106) stimuli are found in persons with prior mTBI. In addition, altered central sensory processing of vestibular (107, 108), visual, (101, 107, 108), and auditory (109) signals has been proposed as underlying persistent mTBI-related symptoms, including those indicating cognitive dysfunction. Moreover, multimodal sensory management requirements incur a greater processing demand for persons with prior mTBI (110–112). Compromised multimodal sensory management of visual-vestibular stimuli is commonly found in persons with mTBI (110–113). The added processing challenges of multimodal signals often leads to impaired cognition well past the onset of mTBI. For example, dual visual-auditory processing deficits have been reported more than 2 years after mTBI, and results in greater deficits in cognition including executive dysfunction (114).

As in MS, thalamic pathology occurs in mTBI, and further exacerbates impaired sensory processing and cognitive dysfunction. Reductions in thalamic and hippocampal volume have been reported in mTBI 1 month after injury, and at 6 months post-injury, volume loss persists in the thalamus only (115). Moreover, it was found that the 6-month thalamic volume loss correlated with persistent vestibular and cognitive symptoms (115). Reduced processing speed and working memory were found in persons with mTBI who had symptoms lasting 32 days post-injury, and impaired working memory correlated with decreased thalamic functional connectivity (116).

Also similar to the pathology of MS, damage to WM connections of the thalamus is important in mTBI. Bai et al. recently showed that impaired processing speed and working memory are persistent problems up to 6–12 months post-injury, and relate to WM disruption most notable in the thalamo-cortical radiations and the anterior corpus callosum (117). Consistent with recent findings in MS (89), it has been proposed in mTBI that WM tract damage may lead to progressive GM degeneration in the thalamus, further perpetuating chronic post-mTBI cognitive impairment (118, 119).

Taken together, a growing body of evidence from studies of MS and mTBI underscores the burgeoning link between disrupted primary sensory processing and cognitive dysfunction. White matter damage plays a central role in this relationship, either through overt injury or more subtle loss of WM integrity. In these disorders, cognitive dysfunction may initially be related to slowed signaling in relevant WM tracts, and, in some cases, GM pathology secondary to WM disruption in networks responsible for sensory processing and cognition. Added to this problem is the challenge of concurrently managing multimodal sensory stimuli, which can lead to processing demand overload and interference with normal cognitive processing. This sensory overload, or sensory processing flooding effect, can be found in cognitively unimpaired persons (120) and in diseases such as schizophrenia (121), but is magnified in disorders such as MS and mTBI that feature pathological sensory processing and cognitive impairment resulting from damaged WM.

## Discussion

By virtue of living in the natural world, humans are constantly exposed to environments with various levels of concurrent external stimuli. Sensory input from the visual, auditory, and vestibular systems is required for the brain to process critical information, which then enables a variety of psychological and behavioral responses. Successful processing of multimodal primary sensory signals thus serves as a necessary condition for normal cognitive function. These signals provide a foundation for human behavior, which then manifests as a complex amalgam of cognitive and emotional domains that requires bi-directional data exchange within distributed neural networks for long-term development and modulation.

This review highlights the role of primary sensory input in cognition by considering the visual, auditory, and vestibular modalities. Although the thalamus is a key sensory data hub in these processes, numerous WM tracts such as the thalamic radiations, cingulum, and uncinate fasciculus are crucial for the relay of initially processed sensory signals to cortical regions for higher-level coding that enables both bottom-up and top-down processing. In addition, the WM tracts of the middle and superior cerebellar peduncles play critical roles in sensory signal processing and the facilitation of cognitive function. White matter tracts make up the mediating circuitry between GM structures throughout the brain, and the distributed neural networks that include GM and WM comprise the infrastructure for multimodal sensory processing and cognition.

As is observable in disorders such as MS and mTBI, pathology of the thalamus and its associated WM tracts results in cognitive impairment that furthermore can be associated with altered unimodal and multimodal sensory processing. These observations suggest that further research is warranted to better define the causative relationship between disrupted sensory processing and cognitive impairment in these and other disorders, with a special focus on WM pathology. Studies are also needed that investigate differentiating the impact of the initial effects of slowed signaling in tracts and, in some cases, GM pathology secondary to WM disruption, as compared to the flooding effect of sensory processing on cognition. Critical to investigations of this kind will be the application of advanced neuroimaging that will allow for the detailed depiction and analysis of healthy and pathologic WM tracts. To help elucidate subtle findings in WM tracts implicated in the sensory-cognitive connection, more precise imaging of WM microstructure will be necessary. Therefore, in addition to conventional MRI and DTI, future studies will benefit from the deployment of emerging WM imaging techniques and approaches, such as neurite orientation dispersion and density imaging (NODDI) to provide a more detailed and accurate assessment of microstructure than standard metrics such as fractional anisotropy [FA; (122, 123)], and from the application of multiple MR modalities that are sensitive to different neurobiological features such as magnetization transfer saturation, and R1 and R2^*^ metrics that add triangular compliments to routine tractography metrics such as FA to enhance WM specificity (124).

In the clinical setting, standardized neuropsychological assessment of cognition is important and useful, but it has some limitations when multisensory dysfunction is considered. Current cognitive assessment measures are effective for detecting common and overt cognitive impairments; however, they are less useful for identifying covert dysfunction, and determining functional, contextual factors that implicate real-life, ecological considerations such as sensory processing demands that contribute to cognitive impairment (125). This point is illustrated by the directive that, for diseases such as MS and mTBI, the standard administration of cognitive assessments is to be conducted in “quiet”, “sterile” environments “without distractions” (126, 127).

Recent reviews of neuropsychological testing have suggested a transition from a reductionistic approach (e.g., focusing on strict association between localized brain pathology and cognition, and assessing within a decontextualized environments) to a more inclusive, real-world approach (128, 129) that accounts for naturalistic multimodal sensory processing (130, 131). Such strategies would enhance the early detection of both the initial pathological alternations in sensory processing, and the additional multimodal sensory flooding effect on cognition, and are likely to disclose more subtle cognitive loss, especially in early disease stages when prompt treatment may lead to better outcomes (132).

Evidence gathered from studies discussed above will better inform researchers and clinicians so that more precise therapies can be investigated for their effectiveness. Attention should be devoted to interdisciplinary care or integrated practice units, where cognition can be considered a shared outcome so that therapies informed by the WM contributions to cognitive impairment discussed in this review can be implemented. Among the various disciplines offering care (e.g., rehabilitation, psychology), the use of experiential therapy approaches, that require managing enriched environments and novel multimodal sensory signals, can aid in providing the appropriate stimuli required for augmenting experience-dependent brain activity, neuromodulation, plasticity, and ultimately cognitive function (133–135). The application of such therapies would be intended to exploit the potential for dual targeting of cognitive loss in WM disorders, from pathology in WM tracts subserving cognition, and from sensory overload resulting from altered multimodal processing that further interferes with cognition.

## Conclusion

The contributions to cognitive impairment made by altered multimodal processing of primary sensory stimuli represents an important perspective in behavioral neurology. Whereas, agnosias and related syndromes have long been appreciated in association with pathology in higher sensory regions of the brain (25), primary sensory dysfunction and its relationship to cognitive impairment has been more recently recognized and investigated. In this review, two disorders with prominent WM pathology have been discussed to point out that impaired cognition can occur not only because of involvement of cognitively relevant regions, but also because of simultaneous processing of primary visual, auditory, and vestibular stimuli that can overwhelm the capacity for normal engagement of those regions. The interference with normal cognition that can result from the burden of multimodal sensory processing illustrates how altered WM connectivity of primary sensory systems plays a key role in cognitive dysfunction. This insight adds more nuanced detail to the behavioral neurology of WM (136, 137), and suggests that a deeper understanding of the concept of WM dementia (138) may be achieved by considering tracts not typically considered relevant to cognition. Supporting this approach, innovative spatially unbiased region and network-based methods such as the Network Modification (NeMo) Tool developed by Kuceyeski et al., a novel topology software pipeline, should be considered to augment the ability to predict GM connectivity outcomes based on changes in WM in diseases such as MS (139). The cognitive impact of abnormal multimodal sensory integration from WM pathology offers a host of intriguing opportunities for further research to investigate fundamental WM-behavior relationships, develop more sensitive clinical testing instruments, and seek improved treatments for a wide range of WM disorders.

## Author contributions

Both authors listed have made a substantial, direct, and intellectual contribution to the work and approved it for publication.

## Conflict of interest

The authors declare that the research was conducted in the absence of any commercial or financial relationships that could be construed as a potential conflict of interest.

## Publisher's note

All claims expressed in this article are solely those of the authors and do not necessarily represent those of their affiliated organizations, or those of the publisher, the editors and the reviewers. Any product that may be evaluated in this article, or claim that may be made by its manufacturer, is not guaranteed or endorsed by the publisher.
